# Exploring the Potential of Technology to Promote Exercise Snacking for Older Adults Who Are Prefrail in the Home Setting: User-Centered Design Study

**DOI:** 10.2196/41810

**Published:** 2023-05-24

**Authors:** Katarzyna Stawarz, Ian Ju Liang, Lyndsay Alexander, Angela Carlin, Anjana Wijekoon, Max J Western

**Affiliations:** 1 School of Computer Science and Informatics Cardiff University Cardiff United Kingdom; 2 Department for Health University of Bath Bath United Kingdom; 3 School of Health Sciences Robert Gordon University Aberdeen Aberdeen United Kingdom; 4 Centre for Exercise Medicine Physical Activity and Health, Sports and Exercise Sciences Research Institute University of Ulster Newtownabbey United Kingdom; 5 School of Computing Robert Gordon University Aberdeen Aberdeen United Kingdom

**Keywords:** physical activity, older adults, Internet of Things, user-centered design, qualitative research, mobile phone

## Abstract

**Background:**

Older adults are at increased risk of falls, injury, and hospitalization. Maintaining or increasing participation in physical activity during older age can prevent some of the age-related declines in physical functioning that contribute to loss of independence and low reported quality of life. *Exercise snacking* may overcome some commonly cited barriers to exercise and encourage older adults to engage in muscle strength and balance activity, but the best way to deliver and support this novel format remains unknown.

**Objective:**

Our aim was to explore how the novel *exercise snacking* approach, that is, incorporating short bouts of strength and balance activities into everyday routines, could be supported by technology within a home setting and what types of technologies would be acceptable for older adults who are prefrail.

**Methods:**

Following a user-centered design process, 2 design workshops (study 1) were conducted first to understand older adults’ (n=11; aged 69-89 years) attitudes toward technology aimed at supporting exercise snacking at home and to inform the design of 2 prototypes. Next, based on the findings of study 1, an exploratory pilot study (study 2) was conducted over 1 day with 2 prototypes (n=5; aged 69-80 years) at the participants’ homes. Participants were interviewed over the telephone afterward about their experience. Transcripts were analyzed using framework analysis.

**Results:**

The results showed that the participants were positive toward using technology at home to support exercise snacking, but both exercises and technology would need to be simple and match the participants’ everyday routines. Workshop discussions (study 1) led to the design of 2 prototypes using a pressure mat to support resistance and balance exercises. The exploratory pilot study (study 2) participants reported the potential in using smart devices to support exercise snacking, but the design of the initial prototypes influenced the participants’ attitudes toward them. It also hampered the acceptability of these initial versions and highlighted the challenges in fitting exercise snacking into everyday life.

**Conclusions:**

Older adults were positive about using technology in their homes to support strength and balance exercise snacking. However, although promising, the initial prototypes require further refinement and optimization before feasibility, acceptability, and efficacy testing. Technologies to support exercise snacking need to be adaptable and personalized to individuals, to ensure that users are *snacking* on balance and strengthening exercises that are appropriate for them.

## Introduction

### Background

The benefits of physical activity (PA) across the life span are well documented [1]. Within the United Kingdom, older adults (aged ≥65 years) should accumulate 150 minutes per week of moderate-intensity aerobic activity [2]. Furthermore, the guidelines highlight that any level of PA should be encouraged, and activities to improve or maintain muscle strength and flexibility should be incorporated at least 2 days per week [2]. However, many older adults are failing to meet these guidelines and report low levels of muscle and bone strengthening activities [3]. Older adults are at increased risk of falls and injury owing to age-related declines in physiological functioning [4], which can impede their quality of life and independence and place an enormous strain on health and social care costs at the societal level [5].

Recent studies have also indicated that older adults spend a high proportion of their day engaged in sedentary behaviors [6], that is, any waking activity in a sitting, lying, or reclining posture where energy expenditure is <1.5 metabolic equivalents [7]. As the proportion of older adults in our society increases [8], strategies to promote PA and reduce sedentary behavior in this age group are important to maintain physical functioning [9] and improve health-related quality of life [10].

To promote and sustain participation in strength and balance exercise as individuals age, there is a need to develop interventions for this population that are effective, inclusive, acceptable, and safe [11]. Furthermore, interventions should enable older adults to overcome some of the commonly cited barriers to current participation in PA. Such barriers include dislike for activities that are structured or sport based, time commitments, and limited access to facilities [12-14]. Integration of functional exercise into daily routines may provide another alternative to PA promotion in this population and overcome the recognized barriers in relation to structured exercise programs [15].

Incorporating short bouts of exercise across the day or *exercise snacking* [16] represents an innovative approach to PA promotion among older adults. It is similar to Snacktivity [17,18], which is mostly used in the context of aerobic PA*.* Both promote opportunities to engage in exercises that are safe and compatible with individuals’ surroundings and lifestyle [16]. So far, exercise snacking has been shown to be an accessible, acceptable, and effective alternative to traditional exercise in older adults [16,19].

Technology has the potential to support PA at home. Recent studies have focused on wearables and activity trackers such as Fitbit [20], which can be effective in encouraging PA and reducing sedentary behaviors [21]. However, these devices tend to focus on supervising or monitoring older adults and tend to support a limited number of activities, especially cardiovascular activities such as walking [20]. Given their reliance on measuring steps and location, they are unsuitable for supporting strength and balance exercises. Similarly, previous studies on supporting older adults’ exercise at home have focused on more complex solutions such as Kinect [22] or social robots to support (predominantly aerobic) PA [23]. These solutions are expensive and require planning to fit the exercise sessions into one’s day. Owing to the situated nature of exercise snacking and its links with everyday routines, Internet of Things (IoT) devices are well suited to provide technological support. IoT devices can be easily embedded at home and provide both monitoring and guidance, such as reducing office workers’ sedentary behavior [24], supporting good posture while sitting [25], or exercising [26]. As such, they could be used to support exercise snacking at home as part of routine everyday activities.

### Objectives

This project explored how ubiquitous technology could be embedded in the home setting to support community-dwelling older adults who are prefrail with exercise snacking activities to improve strength and balance. It builds on previous studies that have demonstrated exercise snacking to be as effective as resistance training in improving muscle functioning [16] but has the added benefit of overcoming barriers to engagement in PA for older adults.

The main objective was to develop and test a set of interactive prototypes that could be embedded in the home environment to support strength and balance exercises. To do so, we engaged older adults who are prefrail in the design of the prototypes and conducted an exploratory home evaluation. Health technologies tend to be designed without consideration of older adults’ perspectives about PA [27], which can reduce their usability or adoption within this user group. Therefore, our goal was to work directly with older adults and use their input and ideas as a starting point to ensure that the prototypes addressed their needs.

## Methods

### Approach

This project followed an iterative, user-centered design (UCD) process [28] to identify the requirements for initial prototypes and explore their potential; however, we did keep in mind the principles of person-based approach [29], as this work will be used as a starting point for the development of a future behavior change intervention. Study 1 (design workshops) aimed to identify appropriate exercises that older adults are willing to do at home and attitudes and preferences toward technologies that might support PA. Study 2 (home evaluation) then developed and piloted new prototype technology informed by the results of study 1.

### Participants and Recruitment

We recruited 16 community-dwelling older adults who are prefrail from participants of a randomized controlled trial (focused on encouraging PA among older adults [30]) who consented to be approached for future research projects. Of the 16 individuals, 11 (69%) participated in study 1 (mean age 74, SD 5.5; range 69-89 years). Of the 11 participants, 7 (64%) were women and all (n=11, 100%) were White British. A further 5 participants participated in study 2 (mean age 74, SD 4.87; range 69-80 years), and 3 (60%) of them were women. Participants who responded to the study email advertisements were sent a participant information sheet describing the study.

### Study 1—Design Workshops

#### Materials

Study 1 involved two 2-hour design workshops in Bristol, the United Kingdom. To facilitate discussions, participants were provided with handouts showing examples of specific muscle strengthening and balance exercises and simple Tai Chi movements they could do at home, which were also demonstrated to participants by a trained exercise instructor (IJL). A set of electronic components (eg, proximity sensors, pressure mats, vibrating components, and lights), examples of wearable devices (eg, a smartwatch and an activity tracker), and an Amazon Alexa were used to facilitate the discussions about technology supporting exercise snacking at home.

#### Procedures

Workshops were conducted on the same day in February 2020 within a Sensor Platform for Healthcare in a Residential Environment (SPHERE) smart home [31]. The smart home belongs to the University of Bristol and is a terraced house with several rooms equipped with various sensors such as movement sensors and near-field communication (NFC) tags. The sensors were visible throughout the home; however, they were not used as part of this study. Nevertheless, participants were able to see how smart technologies could be implemented in a home environment, which facilitated discussions about how new devices could fit into their existing homes.

Each session started by discussing the participants’ current PA levels, including home-based and group activities and any barriers to exercise that they had encountered. Exercise snacking and exercise handouts (preferred format and content) were then discussed. A researcher and trained exercise instructor demonstrated 5 exercise snacking and 5 Tai Chi snacking movements [19], with participants trying each. This session was conducted in a living room, and participants were able to use chairs and a sofa as part of the exercises. Participants then discussed their thoughts about the exercises and how they could be fit into their daily routines and home environments.

Participants then moved through the house (kitchen, bathroom, bedroom, and dining room), discussing suitable exercises for each room, how rooms differed from their own environment, and how that difference could affect the exercise. In addition, any technology that could support and prompt exercise was discussed. This was facilitated by a member of the research team. Subsequently, 1 researcher presented examples of various technologies and sensors, explaining how each item worked and how it could be used in practice. Participants then discussed which components and devices could be useful to support exercise snacking in their home environment.

After the study, participants received a shopping voucher worth £10 (US $12.91) for participating in the workshop.

### Study 2—Feasibility Evaluation With Semistructured Interviews

#### Materials

**Figure 1:**
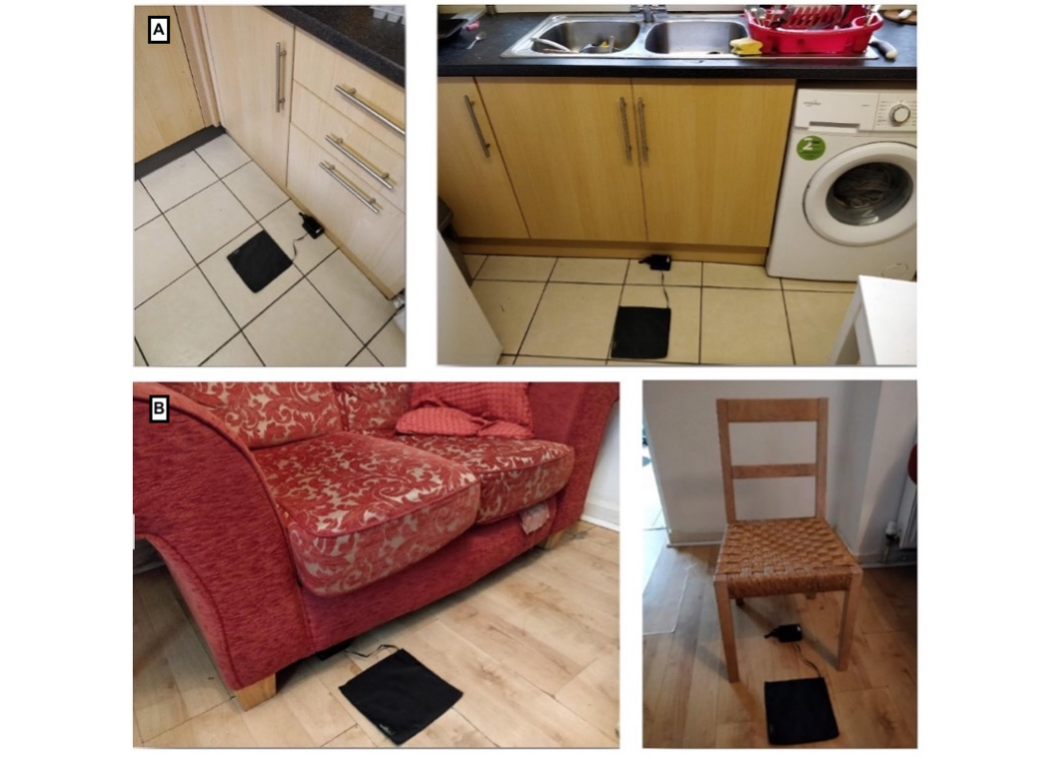
Exercise snacking prototypes for supporting (A) 1-leg balance and (B) sit-to-stand exercises. Images were captured by the researchers to demonstrate potential locations for the prototypes.

On the basis of the key findings from study 1, a total of 2 types of interactive prototypes were built: 1 to support 1-legged balance exercises and 1 to support sit-to-stand exercises (Figure 1). These 2 activities were chosen because participants agreed that they were useful and were the easiest to be integrated into their everyday routines, that is, were easy to master and could be done anywhere at home. Each prototype consisted of a pressure mat and a companion screen. As their design was influenced by study-1 results, more details are provided in the *Prototype Development* section after study-1 results.

The prototypes were accompanied by a booklet explaining exercise snacking and the 2 selected exercises, with advice about how to do them correctly and suggestions of times and places at home where they could be done. The booklet also included a setup and troubleshooting guide.

#### Procedures

The evaluation study consisted of 2 parts (an exploratory home evaluation and an interview) and was conducted in Bath, the United Kingdom, between May 2021 and June 2021. Written informed consent was obtained from all participants. No personally identifying information was collected and the data were anonymized. Prototypes were delivered to participants’ homes by a researcher; participants also received written setup instructions, and the researcher was available via telephone to provide any further technical and exercise support. Owing to COVID-19 restrictions, the researcher followed a COVID-19–secure process, which involved contactless delivery and collection, with no entry into the participant’s home. Each participant received both prototypes (balance mat and sit-to-stand mat) and was requested to use them for a single day. They were asked to think about their everyday routines and place the prototypes and the feedback screen in spaces where they would be the most likely to see and use them without having to go to a dedicated room. After delivering the prototypes, the researcher explained each exercise to the participant over the telephone.

At the end of the day, a researcher collected the prototypes and, later, conducted the telephone interview. Interviews lasted approximately 30 to 45 minutes and covered participants’ general experience of setting up and using the prototypes, views about the utility of the technology going forward, and their general views about how to improve the prototypes or better integrate technology into their daily lives. After that, participants received a shopping voucher worth £30 (US $41.67) for testing the prototypes at home and participating in the phone interviews.

### Ethics Approval

Both studies received favorable ethical opinion from the University of Bristol (project ID 99482) and Cardiff University (COMSC/Ethics/2020/071).

### Data Analysis

Study-1 workshop discussions were audio recorded and transcribed verbatim for subsequent analysis. Any mention of participants’ names in the transcripts was replaced by participant numbers before the analysis. Transcripts were analyzed thematically [32] using both deductive and inductive approaches to explore insights related to the specific topics we focused on and any unexpected findings. Before analysis, transcripts were read to identify specific features for the prototypes, so that they could be incorporated by the developer while data analysis continued. One of the authors (IJL) coded all the transcripts. Codes were then reviewed and discussed by 2 other authors (KS and MW), who identified the provisional themes and drafted the results. The themes and draft findings were then reviewed and discussed with all authors until the final themes were fully defined.

Study 2 ended with semistructured telephone interviews, which were audio recorded and transcribed verbatim. Transcripts were anonymized. Framework analysis [33] was applied to the data, as the authors were interested in specific topics. Following familiarization and coding of the transcripts, one of the authors (AW) created a framework table using interview questions as categories (columns), and each participant was allocated a row, with codes in corresponding cells. Then, 2 authors (AW and KS) summarized the findings in each cell to identify potential themes. Provisional themes were drafted by 2 authors (KS and MW) and then discussed with all authors, leading to the strengthening of some themes and removal of others.

## Results

### Study 1—Workshops

#### Overview

We were interested in understanding participants’ views about and attitudes toward exercise, PA at home, and technology—these discussion topics formed the initial structure for resulting themes. Within each topic, themes and subthemes identified through the analysis are reported. They are summarized with representative quotes in Multimedia Appendix 1 and described in more detail in the following sections.

#### Topic 1—Attitudes Toward Exercise

Several themes were identified in relation to common *barriers to participation*. A common point of discussion centered around leisure settings being viewed as a nonwelcoming space for older adults. Several participants pointed to leisure centers and gyms as being young and male dominant, whereas others recognized that much of the provision for older adults was group based, with participants noting that they felt a lack of confidence in exercising with others. There were also barriers relating to individuals’ motivation to do regular exercise. This was linked to the fear of falling and injury or lack of baseline strength, which made exercise a perceived risky prospect. However, despite these participation barriers, there was a strong sense that *exercise was important to the participants,* with many highlighting the health and well-being benefits it brings. Participants also recognized exercise to be a way of building confidence to stay engaged in other forms of social and leisure activity. Similarly, the social aspect of exercise itself was regarded as a key driver for participation, particularly for walking and aerobic activities.

Participants generally agreed that exercise should match the profile and ability of the target user and saw *potential in* *exercise snacking* to overcome this issue. For example, there were exercises that were much more suited to people in their later life, particularly owing to their physical capabilities, such as balance or sit-to-stand exercise. In this regard, the exercise snacking concept was viewed favorably, as it was seen to enable people to build up from different baselines and progress on their own terms and appeared to be easy to master as a set of exercises. In addition, it could help to overcome other barriers that participants had mentioned—including the ability to do PA in a low-risk environment that was not a leisure setting. Tai Chi movements, as a proposed format of exercise snacking, had more of a mixed reception, with preconceptions both acknowledging it as a useful, relaxing exercise but potentially tricky to learn.

#### Topic 2—Exercising in the Home Environment

The second topic explored how the home environment might support or hinder regular engagement in exercise snacking. While walking around the smart home environment, some participants commented about the *impact of location* and how different spaces lend themselves to exercise more than others. For example, it was apparent that the amount of floor space in a room was important for it to be seen as a space to exercise in. Another consideration was the need to work around other people at home, including partners, spouses, grandchildren, or pets. There was also a sense that certain rooms had a particular function that would preclude them from being a place for exercise, such as the dining room.

Much of the conversation about the suitability of spaces to exercise centered on *safety in the home environment*. Having solid objects to hold on and to support balance and stability where necessary was seen as an important consideration, with key examples in the more spacious rooms being kitchen worktops and chairs. In addition, for some formats of exercise, such as balancing, having soft furnishings and carpeted floors would make the environment feel safer than hard spaces.

A final theme related to the home environment was how certain spaces or everyday tasks could be used as opportunistic *contextual cues to prompt exercise snacking*. For example, the lounge was suggested as a good place to be prompted and do exercise, as it is typically the space where older adults would otherwise sit for long periods—as such, it would be suitable for exercises that can be done while sitting. Some people identified everyday actions that could prompt their exercise snacking, such as brushing teeth, boiling the kettle, or washing dishes. As they were part of the routine and usually occurred in the same spot, they could be linked with exercises suitable for that space, for example, balance exercises.

#### Topic 3—Opportunities and Challenges of Using Technology at Home

Finally, when discussing the use of technology, it was apparent that participants were already familiar with a range of technologies (eg, apps, Amazon Alexa, Fitbit for tracking steps, and YouTube videos to support exercise) and referred to existing solutions to highlight their strengths and weaknesses. On the basis of this previous experience, they had clear *expectations* of what technologies would work and would not work for them. For example, they all agreed that any system that aims to support PA at home should be discreet or even hidden, as not everyone felt comfortable “advertising” with technology that they were trying to be more active. In addition, such systems should also work for people with low technology literacy and be as simple and easy to use and set up as possible. As such, some participants also thought that limiting functionality would help to make the technology easy to use.

This need for simplicity was also linked with a *need to consider the context of use*. This included accounting for the realities of everyday life and characteristics of the users. A participant mentioned that ideal technology would be something they could use without having to wear glasses. Another participant pointed out that the technology would be a part of a wide ecosystem and, therefore, would need to easily connect to the local wireless network and work with other devices at home. Furthermore, it should be inexpensive, as even smartphones or smartwatches were seen as being beyond the reach of a regular person.

Participants also identified several *opportunities for exercise snacking technologies*. They agreed that technology should provide instructions, feedback, and reminders. Instructions were seen as an important feature that could help to introduce the correct movements; help users understand how to exercise (eg, frequency and when to stop); and later, help check whether they were exercising correctly, especially if no additional support was provided. Regarding the latter point, participants expressed a desire to have access to either support groups or someone they could discuss their progress with. Furthermore, visual prompts could also be used to provide ongoing feedback and situated instructions, for example, by showing the movements one is supposed to execute or simply providing encouragement to motivate the user. In addition, some participants thought that this type of interaction could be more playful and “witty.” Overall, participants were open to trying new technologies. Having identified the best locations and types of exercises, they also suggested building devices that could be incorporated into everyday objects to encourage exercise snacking in a specific location.

### Prototype Development

Study-1 results informed the design of the prototypes. Given that participants expressed interest in exercise snacking and engaging with simple exercises at home, we decided to develop prototypes based around a pressure mat that could be placed anywhere at home, where it would fit best into participants’ daily routine. This form factor would also allow participants to just use it (stand or sit on it) without having to set things up in preparation, which was another aspect identified by participants. Finally, as participants expressed interest in systems that are discreet or hidden, a light-emitting diode (LED) companion screen was included to provide additional visual feedback that did not explicitly mention exercise.

**Figure 2:**
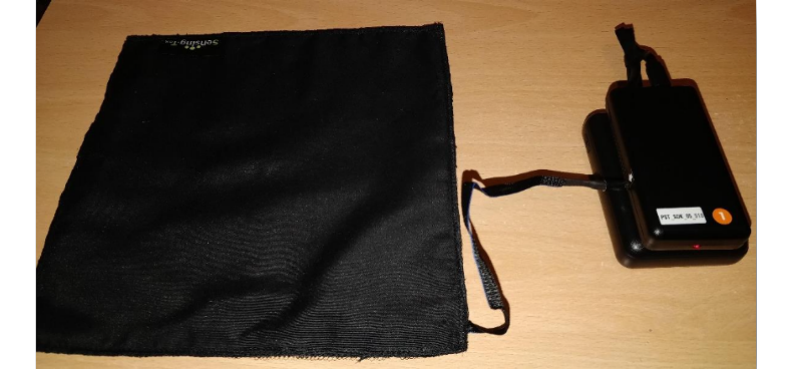
A pressure mat and a battery pack that were used as a basis of the prototypes.

We developed 2 prototypes. Each consisted of a pressure mat and a battery pack. We used SensingTex Switch mat that enabled a single pressure point recognition (Figure 2). We selected a pressure mat as the basis of each prototype, as it would not require any complex interactions, and participants would only need to stand on it if they were ready to exercise. Each mat was connected via Bluetooth to a Raspberry Pi 4 and a Unicorn HAT LED Matrix. The LED screen provided feedback to the user because without it, owing to the mat’s minimal interface, it was not clear whether the prototype was active or whether participants were reaching their goals. The screen also provided encouragement and motivation.

**Figure 3:**
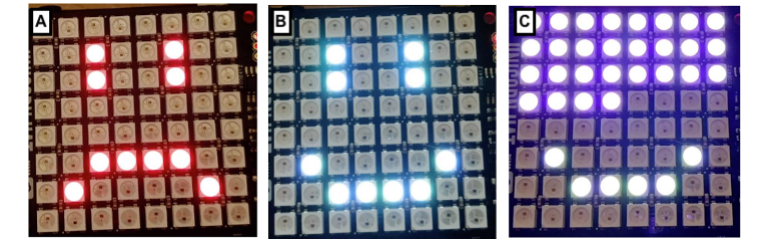
Companion light-emitting diode displays available to the user, showing (A) that the total number of activities for the day has not been reached yet, (B) that the activities for the day have been completed, and (C) a progress bar that fills the screen showing the user how long they should engage with the exercise.

Although both prototypes appeared similar, they had different underlying algorithms to account for differences in the exercises. The balance mat was set up for 2 daily repetitions, 60 seconds each, as a default, and the sit-to-stand mat was set up to measure movement repetitions for 60 seconds during each exercise session, with the target of 30 repetitions per day. When stepped on, the prototype would trigger a timer, which was shown on the LED screen. The devices recorded the number of repetitions and time spent on each exercise. Figure 3 shows examples of the visual feedback—a smiling face if the daily target has been reached, a frowning face if it has not been reached yet, and a progress bar to help count down time during an exercise session.

A flow diagram showing how the prototypes worked is available in Multimedia Appendix 2.

### Study 2—Home Evaluation

The second study focused on evaluating the prototypes at home. Unlike study 1, it was more exploratory in nature—we did not have predefined topics in mind beyond understanding how the participants used the prototypes and what they thought about them.

#### Participants’ Use of Technology

All participants (5/5, 100%) reported using the internet and having previous experience in using mobile phones, laptops, PCs, and tablets. Consumer electronics they used were often Apple products, the sleek design of which was referenced by participants when discussing the study prototypes. Most participants (4/5, 80%) reported that they used some type of activity tracker (often step counters on their phone) and were generally positive toward these types of technologies. Of the 5 participants, 1 (20%) participant mentioned using a heart rate monitor and 2 (40%) reported watching exercise videos on YouTube (fitness and yoga) during the COVID-19 lockdowns to help them stay active.

These technologies and participants’ experiences influenced how they interacted with our prototypes and their expectations toward the devices. In the following sections, we describe 4 themes identified in the interviews conducted after the home evaluation.

#### Importance of Design Esthetics and Reliability

The study findings highlighted the importance of selecting the right level of complexity and polishing the design of initial prototypes used for testing. As the study’s goal was to explore how the technology fits into people’s homes and could support exercise snacking, we focused primarily on the functionality and did not prioritize the design at this stage. Therefore, our participants thought the prototypes were crude (“The graphics I thought were very crude. I think they could have been more pixels in the display to make the pictures easier to understand.” [Participant 5]) and unfinished (“That equipment was quite awkward, you know, the cables and the fittings and the plugs didn’t seem to fit very securely. It was all kind of it all looked a bit fragile.” [Participant 3]), which affected how they used them. Some participants were not sure whether they could fold the mats for storage or whether that would damage them.

Participants also found it cumbersome to assemble the prototypes and to remember to switch them on and off to preserve the battery. This led to technical issues when they connected things incorrectly or the prototypes were not working properly, which discouraged participants from using the devices. Therefore, although participants agreed that the devices had potential and “tools like this” could be useful, they did not see a clear benefit of using the prototypes in their current form:

The technology is too crude and intrusive at this early stage, compared with either a) doing without or b) doing something clever with it.

Participant 1

#### Challenges With Fitting the Device Into Everyday Life

The prototypes were designed such that they could be used in different places at home to enable participants to fit exercise snacking into their routines. Although it mostly worked, participants highlighted a few practical considerations. A participant reported that they had to rearrange their house and move a chair to the kitchen so that they could do the sit-to-stand exercise:

I’ve got lots of things all over the place. For this trial, I put them in the kitchen. But it would be in the way if I left there every day. I’d have to find somewhere else.

Participant 1

In addition, the balance mat was perceived as a potential trip hazard and participants were not keen to leave it on the floor when not in use:

If I kept it there during the day, I could easily trip on it. Anybody could trip or slip as well because I have a wooden floor so it could slip quite easily.

Participant 2

In addition, some did not want the prototypes to always be visible owing to their looks and found packing and unpacking the devices to be cumbersome. Similarly, the limited battery life required the device to be switched off when not in use, which added an extra burden.

Overall, although exercise snacking was supposed to be easy and effortless, having to set things up defeated the purpose of the prototypes. Participants acknowledged that although having a dedicated tool could, in principle, make exercising easy, using preexisting methods or devices was perceived as easy and more useful. Improved reliability, more polished look, and easy set up would make exercise snacking systems more motivating and appealing:

I think [the prototypes] just feel, um, they don’t feel user friendly and they don’t feel...they feel like old technology. I think it would need to have a screen; it would need to look like a phone; it would need to have a digital reader, you know, all of that, like the apps we have on our phone.

Participant 3

#### Need for Personalization

The initial design of the prototypes allowed changes to the difficulty levels and the number of repetitions. However, in scaling down the project owing to the COVID-19 pandemic, the researcher gave all participants (5/5, 100%) the same device with default settings. This proved to be problematic, and participants consistently commented that the exercises were either very easy or very difficult:

The balance exercise is quite easy, and the sit-to-stand is more strenuous. It’s hard work. It takes more energy and makes me tired.

Participant 5

A positive side effect observed was increased motivation of a participant who started using weights to make the exercises more challenging (“I put a rucksack on my back and weights in it and I did it like that to get myself...to make [sit-to-stand exercises] harder.” [Participant 3]), which suggested that the device could be useful for initiating exercise behavior or as a gateway to people forming a new routine:

If it’s there, you’ll use it. And that’s just getting into the regime, it’s like, in the morning, you'll sort of do 10 minutes of different exercises...And it just becomes a habit.

Participant 4

Issues with difficulty levels and progression led to discussions about exercise personalization and suggestions for future improvements. Participants believed that exercises needed to be adapted to the user and therefore suggested including some progression to keep users engaged, for example, by increasing the complexity or difficulty of movements:

Trick is to make it sufficiently interesting and challenging to those who find it fairly easy, but also not to put off people who find it harder and struggle to get out of the chair, so maybe if you had a series of levels so you could come in at level one or you could jump to level three.

Participant 3

They also highlighted the need for feedback about the movements and progress, which would help with motivation and could support the increasing difficulty levels (a participant suggested a potential app similar to the Couch to 5K running program but for strength and balance).

#### Future Opportunities

When asked for views about the potential of technology to support home-based exercise after using the prototypes, participants identified several desirable features to improve utility. Features included linking of the devices to a more sophisticated app or sensors to provide more detailed feedback (“If the mat sensed the growing extent of my imbalance and reduced the time, or increased it if my balance was perfect, that would be a bit more useful, and if it sensed where my toes or heel or whatever was going wrong, and issued warnings about posture then that would make it more useful.” [Participant 1]), adding voice or sound as a way of providing feedback about performance of exercises and encouragement, and prompts and reminders to do the exercise.

Participants also discussed how the prototypes could be improved. Some suggestions focused more on how the mat could support a more diverse array of exercises:

It would be nice to have a wider range of exercises. I mean, as you get old your backs get stiff. You get stiffness in lots of joints. I think that it could be done to use more joints as a body, try and create more flexibility.

Participant 5

Participants also provided positive perspectives about the role of technology in supporting home-based exercise, for example, providing visual prompts:

The little pad would be sitting on the floor, would be a reminder.

Participant 5

Others discussed how functioning technology could provide structure to support current activity and could be useful for engaging people in new activities in the short term, even if not used continuously:

At the moment I do it when I can see I’ve got two or three minutes to do sit to stand. So I do.

Participant 1

## Discussion

### Principal Findings

#### Overview

The aim of this project was to develop and test interactive prototypes to be used at home to support strength and balance exercise snacking in older adults who are prefrail. Our workshops identified that participants were open to using technology in the home setting, but personalization of the exercise snacking regime and simplicity in technology use are important. Participants who subsequently tested 2 prototypes (balance mat and sit-to-stand mat) in a home evaluation demonstrated that this technology had the potential to support exercise snacking in the home setting with further development and testing. In the following sections, we discuss the main results and implications for designing systems that support exercise snacking at home for older adults who are prefrail.

#### Home Environment as a Space to Exercise

Exercise was identified as an important activity for participants, and using the home setting as a location for exercise snacking elicited both positive and constructive views, which will inform the next iterative step in the design process. The home setting has been frequently used for rehabilitative exercise for multiple conditions such as musculoskeletal joint replacement, neurological conditions, and cardiorespiratory conditions and has been shown to be as effective as supervised or group exercise at 12 weeks on health outcomes for women with type 2 diabetes [34]. A recent pilot randomized controlled trial has demonstrated that resistance exercise snacking is safe and acceptable for community-dwelling older adults over a 4-week period. Although this project focused on only 1 strength exercise and 1 balance exercise, the results align with those of Fyfe et al [35] and Liang et al [19], who found the exercises to be feasible and safe.

#### Role of Technology for Overcoming Barriers

A key challenge for any exercise program, especially those targeting individuals in the home setting, is prolonged adherence [36]. Participants in this study were largely positive about the potential for technology to support the implementation of home-based exercise snacking as long as the technology was simple, reliable, and unobtrusive to use. The need for simplicity and reliability as key guiding principles for the adoption and sustained use of technology has been found in other studies of older adults’ perceptions about technology [37]. Technology, if designed appropriately, also enables the integration of some key behavioral science principles that can help with exercise motivation, such as self-regulatory behavior change techniques (eg, feedback and goal setting) and gamification to make exercise fun and engaging [37-39], and nudges or cues to help turn exercise into a more automatic behavior [40]. Our preliminary evaluation suggested that more work could be done to improve the reliability of the technology, better integrate feedback, and make the device more personalized to the user’s needs and preferences for exercise.

#### Recommendations for Future Practice and Studies

Accordingly, we provide design recommendations for developing home-based systems that support exercise snacking and other types of PA aimed at older adults. Researchers and developers working in this area should do the following:

1. *Support personalization*—As older adults can have varying levels of activity, any home-based system that facilitates and supports exercise needs to be able to accommodate different baseline circumstances, from fully sedentary routines to physically active users who may want to move more at home. As such, systems should allow users to change difficulty levels, which should then progressively adapt based on the user’s progress.
2. *Provide clear and meaningful feedback*—As exercise snacking is a situated activity, any system that supports it needs to recognize and clearly communicate that it has started and notify the user when they can finish, regardless of whether they are doing timed exercises or a specific number of repetitions. It should also notify the users when they reach their goals and show their progress. Furthermore, different feedback modalities need to be considered to improve accessibility and usability through combinations of visual and auditory feedback to support older adults with hearing and visual impairments.
3. *Take the environment into account*—Any system that supports exercise at home needs to be sufficiently flexible, so that people can use it in the most suitable location. Different people have different routines, and the living situation of older adults varies, which makes it impractical to design a one-size-fits-all solution. For example, some people may prefer to *exercise snack* in the living room, whereas others would prefer to do so in the kitchen; ideally, the system should work in both.
4. *Remember the esthetics*—The design of technologies aimed at older adults is often based on a wrong assumption that esthetics do not matter for this user group. However, increasing access to consumer electronics influences the perceptions about technology and people’s expectations and values; older adults are not different [41]. Furthermore, any device that is meant to become part of an environment should fit into that environment and ideally provide subtle and discreet feedback, as not all users may want to advertise to visitors that they are trying to be more active.
5. *Ensure the system is accessible and easy to use*—As older adults’ experiences with technology or digital literacy may be limited, any system aimed at them should have an intuitive design and require minimal setup. Switching technology on and off and selecting user goals and preferences should be implemented in a user-friendly manner that is suitable for the target population. Furthermore, systems should be compatible with the technologies that people already have at home, for example, wireless networks. As most IoT systems rely on the internet connection, the ease of setup and seamless connectivity are key.
6. *Provide guidance to reduce risks*—Finally, any system that supports PA needs to be able to guide the users, as the movements may not be familiar to them or they may require a reminder. This can be directly embedded in the physical system or be provided through a companion app. Regardless of the format, guidance could help to reduce risks of falls, support older users, and educate users about PA.

### Limitations and Future Studies

This was an exploratory project focusing on the early stages of the iterative UCD process and as such had some limitations. Study-1 participant numbers were limited by space and maximum capacity for people in the smart home at the time; however, the numbers were consistent with those in previous design research using workshops to design digital interventions [42]. Study 2 involved 5 participants who used the prototypes for 1 day. As our goal was to assess the usability of the prototypes and gather early feedback, this was sufficient, because usually 4 to 6 participants are required to identify key usability issues [43]. Participants were vocal about issues and constructive suggestions for improvement. Overall, our participant numbers are consistent with UCD studies, and evidence has shown that these methods can provide generalizable design guidelines [44-47].

Participants were recruited from a cohort that had previously participated in a PA trial [48], albeit a mixture of intervention and control participants. Selection bias may have influenced the results obtained, as the participants may have had more positive attitudes toward PA. However, as this was the initial phase in the design process, both benefits of and challenges to developing technology were identified, and future stages in this process (acceptability, feasibility, and efficacy testing) will ensure a wide, representative, and large recruitment of older adults who are prefrail to avoid potential bias [49]. In addition, recruitment from this population enabled participants to reflect about their previous experiences with exercise snacking and provide feedback and suggestions for improvement, which was crucial for this study.

Study 2 was a home study conducted during the COVID-19 pandemic and, consequently, was subject to several deviations from the initial protocol. These limitations influenced the participants’ experiences, which is reflected in the themes. Nevertheless, key lessons were learned about the design and delivery of technology home testing during COVID-19, which can be embedded in the future stages of this project to ensure successful delivery and completion, regardless of whether there is face-to-face or remote delivery. Furthermore, as home testing occurred over a single day, we were unable to evaluate the adherence to exercise snacking. As adherence is crucial to the acceptability and feasibility of the exercise snacking technology design, factors predicting adherence to home-based rehabilitation (intention to engage, self-motivation, self-efficacy, previous adherence, and social support [50]) will be incorporated into subsequent iterations of the design process.

Finally, the design of our prototypes may have influenced the results. Our primary focus was the functionality of the prototypes; we did not fully consider esthetics or visual design at this stage. This resulted in negative comments and, to some degree, affected participants’ interactions with the prototypes. Although we were still able to gather relevant feedback, more polished prototypes and user interfaces would have helped to concentrate the feedback on functionality. As we are following the UCD process, this will be incorporated into the next iterative phase of development of the prototypes.

### Conclusions

Exercise snacking offers a promising approach for incorporating balance and strength PA into older adults’ routines. Our results demonstrated that technology has the potential to support exercise snacking in the home environment for older adults who are prefrail. However, the design of devices not only needs to be easy to use and set up but must also fit into users’ routines and physical spaces. Exercise snacking technology devices also need to be adaptable and personalized to individuals, to ensure that users are *snacking* on balance and strengthening exercises that are appropriate for them.

## Appendix

Multimedia Appendix 1 A table with quotes to illustrate the subthemes identified in the workshops for each discussion topic.

Multimedia Appendix 2 A flow diagram showing how the prototypes work.

## Abbreviations

IoT: Internet of Things
LED: light-emitting diode
NFC: near-field communication
PA: physical activity
SPHERE: Sensor Platform for Healthcare in a Residential Environment
UCD: user-centered design

## Data Availability

The data sets generated and analyzed during this study are available from the corresponding author upon reasonable request.
