# Supplementary material for: Exploring the Potential of Technology to Promote Exercise Snacking for Older Adults Who Are Prefrail in the Home Setting: User-Centered Design Study
Source: JMIR Aging. 2023 May 24;6:e41810. doi: 10.2196/41810 (PMC10248772; doi:10.2196/41810)
Supplement: Multimedia Appendix 1 [file aging_v6i1e41810_app1.pdf]

Appendix 1: A table with quotes to illustrate the subthemes identified in the workshops for each discussion topic.

Topics, themes, and subthemes			*Participant Quotes*
**Attitudes toward exercise**			
	**Barriers to exercise**		
		• Dislike toward leisure settings<br>• Lack of motivation to exercise or laziness<br>• Physical limitation and safety or injury risk	• "I hate going to the gym and getting sweaty. I think it's just horrid, whereas you can do that and build up your strength gradually with that and I thought that was a nice set of exercises."<br>• "I found myself getting lazier and lazier and lazier in the morning because I like reading so it's quite easy for me to sit in bed, get out make a cup of tea, sit in bed with a cup of tea."<br>• "You thought well it's time to do my practice but that means I've got to go and get changed and get into a leotard and [sigh]."<br>• "I did fall off a bike a few years ago which put me off cycling completely and I haven't been on a bike since."
	**Exercise considered as important**		
		• Health and well-being benefits<br>• Build confidence<br>• Social engagement	• "...Being active and doing things can help slow that decline and give you a better quality of life."<br>• "And when you see the results – I mean I already feel the effect of exercises that I've been doing. You know I'm aware my muscles are telling me that I'm making them work and when you see the effect of it on yourself, that's the incentive for me to carry on and just to be fit."

			• "I like the groups as well because they can be quite sociable things as well because there's always the period after when you've had the exercises and things."
	**Potential of exercise snacking**		
		• Need for tailoring to ability<br>• Could overcome barriers to exercise	• "[The exercise should be] age appropriate – somebody actually saying, do it to your own ability rather than you at home thinking, I can do that and then you know, you can't but if you can't find somebody age appropriate to actually do it, you can do that verbal."<br>• "Sometimes you're just relaxing but I think this concept of 'snacking' is very good because it's not like now I have to go and exercise. It's like I'm walking into the kitchen to make a cup of tea and while I'm there, I can do this for a moment or two."
**Exercise in the home environment**			
	**Impact of location**		
		• Amount of floor space<br>• Room function	• "We touched on the kitchen, it might be an option to do things but limited space in terms of houses and I think a big barrier to doing this perhaps could be that you've got to go and shift a coffee table to do an exercise because you got to then do that every time."<br>• "In my experience old people tend to have a load of furniture in their houses."<br>• "The dining room I mean it's not the sort of place, it's not really the sort of room you sort of have to go to do. It's basically where you'd have your meals."
	**Safety**		

		• Objects to hold on to<br>• Soft furnishing	• “That’s the advantage of the kitchen or the bathroom isn’t it, because generally you don’t have – you have a bit of space in the kitchen or the bathroom to do the exercise standing up at the sink or the work surface or whatever and you don’t have to move furniture to do that.”<br>• “The nice thing about [the bedroom] for some of the balance work though is if you unbalance, if you get a little bit unbalanced, if you fall back, you’re on a bed.”
	**Cues to exercise**		
		• Prompt when sitting<br>• Home activities as prompts	• “It could be to make it more habitual and more part of the routine, it is the same place every time, so it is something on the kettle so when you’re doing the tea you’re doing your thing or it is brushing your teeth in the bathroom.”
**Using technology at home**			
	**User expectations**		
		• Need to be discreet<br>• Clear instruction and guidance<br>• Simple to use	• “I think people look at [an activity tracker] and think what’s that old lady doing with wearing one of those! I’d like something much more unobtrusive.”<br>• “I think it’s that you run the risk of doing that and it’s the KISS principle, isn’t it, is the Keep it Simple and therefore the more straightforward and simple it is but if could start going into reams and reams and reams of why you should do this, you switch off a bit, don’t you?”<br>• “For me if this is designed to help you exercise it’s got to be a basic thing.”
	**Need to consider the context of use**		
		• Realities of everyday life<br>• Home technology ecosystem	• “Often like a lounge area, which is often where people maybe do some exercises, there’s a coffee table in the way. They have to shift it and I

			sometimes wonder is it feasible to ask people to have to shift furniture to do these types of exercises? I think that's a little bit of a..."
	**Opportunities for exercise snacking technologies**		
		• Importance of feedback<br>• Need for visual prompts<br>• Technology as part of everyday routines	• "When we had the step counters, I found that quite an incentive to actually go out and do more walking."<br>• "I might want to see at the end of the week how I got on compared to the end of last week."<br>• "I'm not a technology person but something that will pop up with a smiley face and say, hmm, have you bent your knees today or...you know, something like...well, that would work for me, things that would make me smile and laugh."<br>• "[You could have] a bathmat in your bathroom that had that little square black center [the pressure mat] in it, so it was there, you forget it's there but it takes on board information and gives feedback to you about your balance without you having to think about it."
