# Supplementary figures and images for: Exploring the Potential of Technology to Promote Exercise Snacking for Older Adults Who Are Prefrail in the Home Setting: User-Centered Design Study
Source: JMIR Aging. 2023 May 24;6:e41810. doi: 10.2196/41810 (PMC10248772; doi:10.2196/41810)

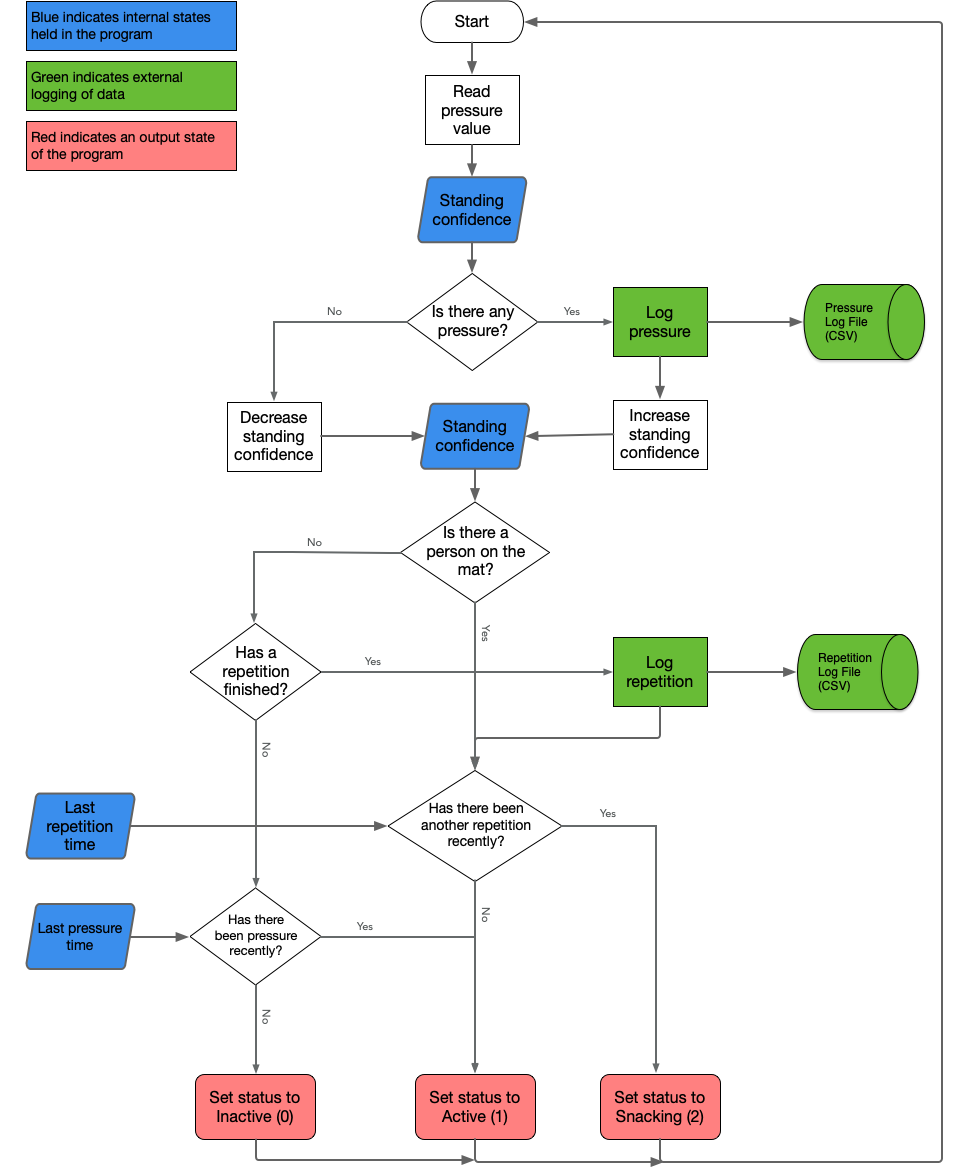

Supplement: Multimedia Appendix 2 [file aging_v6i1e41810_app2.png]
